# Evolution of ungulate mating systems: Integrating social and environmental factors

**DOI:** 10.1002/ece3.6246

**Published:** 2020-04-15

**Authors:** R. Terry Bowyer, Dale R. McCullough, Janet L. Rachlow, Simone Ciuti, Jericho C. Whiting

**Affiliations:** ^1^ Institute of Arctic Biology University of Alaska Fairbanks Fairbanks AK USA; ^2^ Department of Environmental Science, Policy and Management University of California Berkeley CA USA; ^3^ Department of Fish and Wildlife Sciences University of Idaho Moscow ID USA; ^4^ Laboratory of Wildlife Ecology and Behaviour School of Biology and Environmental Science University College Dublin Dublin Ireland; ^5^ Department of Biology Brigham Young University‐Idaho Rexburg ID USA

**Keywords:** mating, monogamy, polygyny, social system, terrain, territoriality

## Abstract

Ungulates exhibit diverse mating systems that range from monogamous pair territories to highly polygynous leks. We review mating systems and behaviors across ungulates and offer a new approach synthesizing how interacting factors may shape those mating systems. Variability exists in mating systems among and within species of ungulates and likely is affected by predation risk, availability of resources (food and mates), habitat structure, and sociality. Ungulate mating systems may be labile as a consequence of the varying strength of those interacting factors. In addition, degree of polygyny and sexual dimorphism in size are associated with the evolution of mating systems. Neither male–male combat nor paternal care, however, can completely explain differences in sexual size dimorphism for ungulates, a necessary component in understanding the development of some mating systems. Whatever the evolutionary pathway, sexual segregation limits paternal care allowing more intense male–male competition. Selection of habitat structure, because it modifies risk of predation, is a major determinant of sociality for ungulates. Likewise, ruggedness and steepness of terrain limit the types of mating systems that can occur because of limitations in group size and cohesiveness, as well as the ability of males to herd even small groups of females effectively. The quality and defensibility of resources affect mating systems, as does the defensibility of females. Population density of females also may be a critical determinant of the types of mating systems that develop. Size of groups likewise constrains the types of mating tactics that males can employ. Our aim was to use those relationships to create a broad conceptual model that predicts how various environmental and social factors interact to structure mating systems in ungulates. This model provides a useful framework for future tests of the roles of both ecological and social conditions in influencing the social systems of ungulates.

## Graphical abstract

We provide a new approach to understanding mating systems in ungulates. We integrate both social and environmental factors in doing so. We develop a predictive model to help understand the evolution of these unique behaviors, which should help to direct future studies.

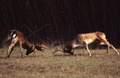

## 1 INTRODUCTION 

**FIGURE 1:**
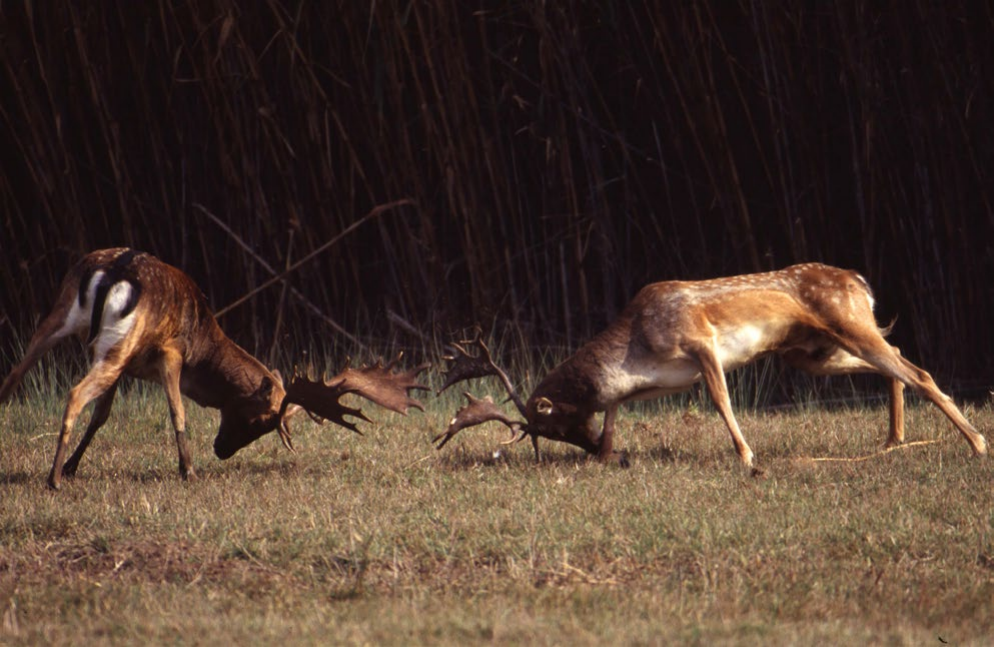
Two fallow bucks (*Dama dama*) fight in the lekking population of San Rossore, Italy, a few days before the peak of autumn rut (Photo by Giuseppe Caleo). Ungulate leks usually occur at traditional sites and are characterized by highly skewed mating success among males (Ciuti et al., 2011)

Ungulates comprise the mammalian order Perissodactyla and include terrestrial members of the Cetartiodactyla (Feldhamer, Drickamer, Vessey, Merritt, & Krajewski, 2015). These hooved mammals exhibit a diverse array of mating systems, which include patterns that range from monogamous pair territories to highly polygynous leks (Apollonio, Cena, Bongi, & Ciuti, 2014; Clutton‐Brock, 1989; Geist, 1974; Jarman, 1983; Lott, 1991; Putman, 1988; Figure 1). This variability exists between and sometimes within species and may be affected by similar elements (Putman & Flueck, 2011; Thirgood, Langbien, & Putman, 2000). Indeed, large herbivores have a unique array of life‐history characteristics that differentiate them from smaller‐bodied taxa (Bowyer, Bleich, Stewart, Whiting, & Monteith, 2014; Caughley & Krebs, 1983). Such differences in life‐history characteristics may constrain and promote the types of mating strategies that evolve. Most reviews of conditions and circumstances fostering the development of various mating systems in ungulates, however, are decades old; we incorporate >100 citations to articles related to ungulate behavioral ecology and mating systems published since the most recent review (Clutton‐Brock, 1989). Moreover, many of those older reviews were primarily descriptive and did not thoroughly integrate both social and environmental influences, although some did discuss social factors affecting mating systems (Clutton‐Brock, 1989). We incorporate and build on those ideas, including using contemporary literature to review and assess previous hypotheses, and developing new premises for the evolution of ungulate mating systems.

Recent studies on ungulates largely have neglected topics such as social behavior and mating systems. For instance, a search with Web of Science^®^ of literature from 2009 to 2018 including the term “ungulate” yielded 4,387 citations. Of those citations, only 4.2% contained the term “social behavior (or behaviour),” and 0.5%, the term “mating system(s).” None provided a broad synthesis of mating systems across the diverse species of ungulates. In addition, many of the older works did not fully consider how habitat and mating behavior interacted to affect the evolution of particular mating systems.

Our aim was to create a conceptual model, based on results from empirical studies, which provides important generalizations about how various environmental and social influences help structure mating systems in ungulates. Mating systems are thought to evolve as a collection of adaptations to environmental and social conditions that benefit individual males and females. For example, similar mating systems, such as tending bonds (discussed later), occur in diverse lineages occupying dissimilar ecological niches, illustrating the need to consider both habitat and social elements in the formation of mating systems in ungulates. Herein, we incorporate older publications with contemporary studies to present an up‐to‐date review of factors contributing to the evolution of mating systems in large herbivores, and propose, to our knowledge, the best likely model that aims to explain much variability in the occurrence of mating systems in ungulates. We provide operational definitions of mating systems and use a broad conceptual approach to arrive at general conclusions concerning mating systems of ungulates. Our model, however, is more than just a simple starting point; we provide benchmarks against which studies of ungulate mating behavior can be compared—our approach should give new impetus to the study of ungulate mating systems.

Factors favoring either group or solitary living lay foundations for the types of mating systems and social behaviors that ultimately evolve (Bertram, 1978; Pulliam & Caraco, 1984). For ungulates, numerous factors are related to gregariousness, including timing of activity patterns, and life‐history attributes such as rut and parturition (Bowyer, McCullough, & Belovsky, 2001; Bowyer, Stewart, Kie, & Gasaway, 2001). The evolution of gregarious behavior, however, can be divided into two broad categories: risk of predation and resources (Bowyer, McCullough, et al., 2001; Bowyer, Stewart, et al., 2001; Jarman, 1974; Putman, 1988). We do not reiterate all potential mechanisms underpinning costs and benefits of living in groups for all mammalian taxa—this issue has been examined previously and is too broad of a topic for our review (Alexander, 1974; Bowyer, McCullough, et al., 2001; Rubenstein, 1978). We note, however, that benefits of group living must exceed its costs for gregariousness to evolve and be maintained. Sufficient costs to sociality or benefits from becoming unsocial will favor a solitary existence. Herein, we briefly recount the manner by which predation and resources affect the degree of sociality and therefore influence mating systems of ungulates.

## 2 FACTORS INFLUENCING GREGARIOUSNESS

### 2.1 Predation

Patterns of antipredator behavior by ungulates serve to reduce probability of detection by predators (e.g., use of concealment cover) or probability of capture (e.g., vigilance, flight, use of escape terrain, or group formation; Bleich, 1999; Caro, 2005; Kruuk, 1972; Molvar & Bowyer, 1994). Risk of predation is hypothesized to affect group size of ungulates via benefits of increased group size in open‐land species that accrue because of more eyes, ears, and noses with which to detect predators at distances that make successful pursuits unlikely (Roberts, 1996). Costs related to competition, however, may be associated with large groups (Uccheddu, Body, Weladji, Holand, & Nieminen, 2015). For forest‐dwelling ungulates, where avoidance of detection is thought to be the primary antipredator strategy, benefits ostensibly ensue from being solitary or living in small groups (Hirth & McCullough, 1977). Open habitat structure promotes large groups, whereas closed habitat results in smaller aggregations (Jhala & Isvaran, 2016). Predation also may affect the number of animals available to form groups by holding ungulates at low density (Gasaway et al., 1992). Even low‐density, predator‐regulated populations, however, can display gregarious behavior (Bowyer, Rachlow, Stewart, & Ballenberghe, 2011; Molvar & Bowyer, 1994). Clearly, conditions can occur where degree of gregariousness reflects more than an adaptive response to changes in habitat structure. Nevertheless, such variation in group size holds import for the types of mating systems exhibited by ungulates.

Ungulates likely communicate information concerning the presence of a predator to other group members through alarm behaviors, including distinctive pelage markings, piloerection of hair, specialized gaits, alarm vocalizations, pheromones, or some combination thereof (Bowyer, Rachlow, Ballenberghe, & Guthrie, 1991; Caro, 1986; Hirth & McCullough, 1977). Those behaviors may help reduce vigilance and improve time spent feeding (Bowyer, McCullough, et al., 2001; Bowyer, Stewart, et al., 2001; Jhala & Isvaran, 2016), including increases in foraging efficiency—that is, percent of active time spent feeding (Berger, 1978). Antipredator behaviors, then, likely fostered other social behaviors related to foraging efficiency that promoted group living in open‐land ungulates. Molvar and Bowyer (1994) demonstrated that social groups of moose (*Alces alces*) formed in response to predation risk without the concomitant benefits of enhanced foraging efficiency, which likely indicates that some benefits of group living may be secondarily evolved. Moreover, sexes of ungulates may use differing tactics to thwart predators—female bighorn sheep (*Ovis canadensis*) use areas close to escape terrain, whereas less‐vulnerable males venture further from such areas; both sexes, however, have similar rates of feeding, vigilance, and foraging efficiency (Schroeder, Bowyer, Bleich, & Stephenson, 2010). Moreover, bighorn sheep move further from escape terrain as group size increases (Berger, 1991)—all those factors are related to sociality and therefore hold potential to affect mating behaviors.

Flight behaviors of ungulate groups have been hypothesized to confuse predators—the juxtaposition of fleeing pronghorn (*Antilocapra americana*) with piloerected rump patches may make the selection of an individual animal to pursue difficult (Kitchen, 1974). Caro (1986) provides additional examples of how “stotting” behavior by ungulates offers similar antipredator advantages. Kruuk (1972) and Schaller (1972) reported that large carnivores, which switch their pursuit from one ungulate to another, have low rates of success. Another benefit of grouping for open‐land species may be an active defense against predators, such as the well‐known defensive stance of muskoxen (*Ovibos moschatus*; Gray, 1987). Sinclair (1977) and Prins (1996) documented aggression by African buffalo (*Syncerus caffer*) toward predators during attacks. Nonetheless, less‐gregarious ungulates that stand their ground against predators also may lower the likelihood of being killed (Bowyer, 1987; Mech, 1970; White, Testa, & Berger, 2001). Ungulates also may harass predators as a defensive strategy (Berger, 1979; Grovenburg, Jenks, Jacques, Klaver, & Swanson, 2009). Pipia et al. (2009) proposed that ungulates may signal the predator that it has been spotted to eliminate advantages of a surprise attack. Similar benefits have been suggested to accrue for white‐tailed deer (*Odocoileus virginianus*) from tail flagging and the subsequent grouping of individuals in more open habitat (Hirth & McCullough, 1977). In those examples, habitat and predation risk combine to affect gregariousness.

Where a predator can capture only a single prey, which often occurs for ungulates and the large carnivores that prey upon them, there may be an additional advantage to grouping. A lone animal has a greater “domain of danger” than individuals in a group and hence a higher probability of being selected as prey than animals occurring in a herd (Hamilton, 1971), termed “dilution effects.” Morton, Haefner, Nugala, Decino, and Mendes (1994) noted that individuals moving toward their nearest neighbor provided an additional antipredator strategy for the “selfish herd.” Ungulates grouping in open country evidently obtain benefits related to risk of predation (McCullough, 1969). There likely are multiple benefits that accrue to open‐land ungulates that live in social groups, and the aforementioned hypotheses are not mutually exclusive (Bowyer, McCullough, et al., 2001; Bowyer, Stewart, et al., 2001). Dehn (1990) reported potential benefits from both vigilance and dilution effects for ungulates occurring in large groups. Putman (1988) postulated that substantial benefits also occur from living in small groups for forest‐dwelling species. Noise and odors from large groups of ungulates moving through dense habitat might interfere with detection of ambush or stocking predators (Bowyer, McCullough, et al., 2001; Bowyer, Stewart, et al., 2001). Indeed, ungulates may vary their group size, vigilance, foraging behavior, and habitat use in response to the hunting style (e.g., ambush or stalking versus coursing) of predators (Atwood, Gese, & Kunkel, 2009; Bowyer, McCullough, et al., 2001; Kohl et al., 2019). In many instances, such variation in degree of sociality may help condition ungulate mating systems.

### 2.2 Resources

**FIGURE 2:**
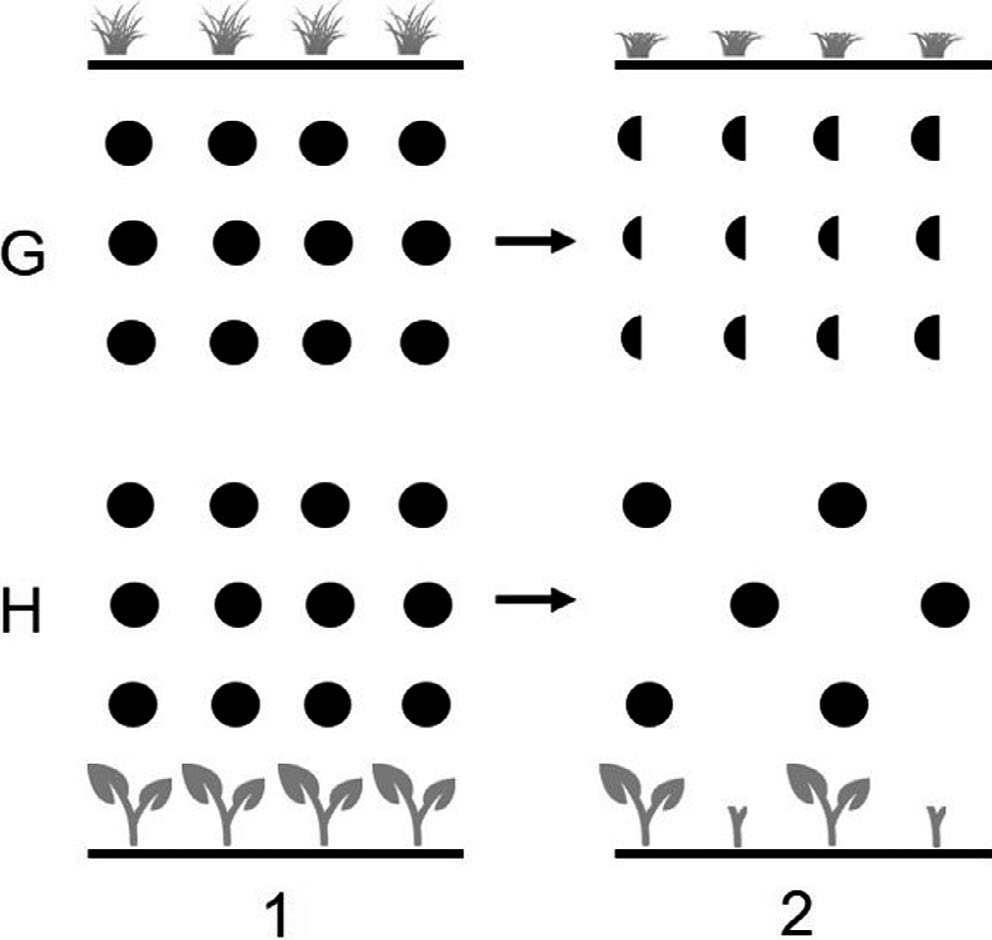
Dispersion of food items before (1) and after (2) one‐half the standing crop is eaten by ungulates. For grasses (G) from which multiple bites may be taken, the dispersion remains the same, but the size of the food item decreases. For herbs or browse (H), whole items are eaten, thereby altering the dispersion but not size of remaining items (from Jarman, 1974)

The distribution and quality of resources influence gregariousness among ungulates. Jarman (1974) proposed that the dispersion of foods affected the degree of sociality. Ungulates that are solitary or live in small groups generally inhabit woodlands, where they selectively forage on dispersed leaves and stems of browse (woody vegetation) or eat herbaceous vegetation (forbs). Ungulates inhabiting open plains, however, occur in large groups. Those ungulates exhibit low selectivity and feed upon more evenly distributed grasses, often in areas with limited forbs and browse. Consequently, Jarman (1974) postulated that in coarse‐grained habitats such as woodlands with a patchy distribution of food items, feeding activities by one animal limit forage availability to others by removing the entire food item (herb, stem, or leaf). Conspecifics would avoid areas where others had foraged, resulting in a wide distribution of animals and a propensity not to form groups. In more fine‐grained habitats, such as open grasslands where food items are more evenly distributed, ungulates remove foods a little at a time; forage is reduced, but the distribution of food items remains relatively constant (Figure 2). Thus, conspecifics could feed closer together and potentially form groups. Groups of large herbivores also may increase productivity of plants and rates of nutrient cycling in areas where they have foraged and deposited urine and feces previously—a process known as “herbivore optimization” (Guernsey, Lohse, & Bowyer, 2015; McNaughton, 1979; Molvar, Ballenberghe, & Bowyer, 1993; Stewart, Bowyer, Ruess, Dick, & Kie, 2006). Ungulates may return to those areas to procure high‐quality foods, which would further promote sociality. Those overall processes provide mechanisms allowing large herbivores to form groups, but fail to explain why they should do so (Bowyer, McCullough, et al., 2001; Bowyer, Stewart, et al., 2001). Moreover, woodlands provide more concealment cover than open grasslands, and changes in sociality with varying degrees of cover also occur (Estes, 1974; Hirth, 1977; Molvar & Bowyer, 1994). The distribution of forages thought to explain interspecific differences in sociality of ungulates (Jarman, 1974) does not hold for seasonal changes in forages in open and wooded areas within some species of large herbivores (Bowyer, McCullough, et al., 2001; Hirth, 1977), where predation risk likely more strongly influences variability in sociality.

The quality and distribution of resources can affect the behavior of ungulates (Belovsky, 1981), although effects of scale on the distribution of resources are important (Bowyer & Kie, 2006; Bowyer, McCullough, et al., 2001; Bowyer, Stewart, et al., 2001). For animals to be social, sufficient resources must exist to allow group living (McNab, 1963), and some threshold for a particular resource may exist at which group formation occurs (Schoener, 1968). For instance, essential resources help set the ecological carrying capacity (*K*), thereby determining the number of large herbivores that a particular area can support (Bowyer et al., 2014; Boyce, 1989; McCullough, 1979). Likewise, heterogeneity of the landscape can determine the size and arrangement of home ranges for large mammals (Kie, Bowyer, Nicholson, Boroski, & Loft, 2002), which also helps govern the number of conspecifics that can associate with one another. Jhala and Isvaran (2016) reported a decline in group size of blackbuck (*Antilope cervicapra*) with increasing patchiness of habitat. Clumped resources can affect the size of social groups and therefore social systems of ungulates.

### 2.3 Mates

In addition to the dispersion of food, availability of mates may influence the size of social groups for polygynous ungulates during the mating season. Large males likely seek out groups of females for mating opportunities. For instance, Bowyer, Bleich, Manteca, Whiting, and Stewart (2007) reported that female American bison (*Bison bison*) were more likely to mate when large males were present compared with groups where only small males occurred, and large males occurred disproportionally in the largest groups. Females also may be drawn to some leks based on the quality of males that hold those territories (discussed later). Mysterud, Coulson, and Stenseth (2002) noted that the presence of males, the age of males, and the ratio of adult males to adult females were related to female fecundity, ovulation date, birth date, and birth synchrony for a variety of ungulates—all outcomes, which under the right circumstances, could result in increased fitness of females and therefore influence grouping behavior. Indeed, males may play a role in the population dynamics of animals (Rankin & Kokko, 2007).

Nonetheless, some ungulates may not experience reproductive benefits postulated for rutting in groups with large males (Mysterud, Langvatn, & Stenseth, 2004; Whiting, Bowyer, & Flinders, 2008). Indeed, the physical condition of females, rather than the characteristics of males, may be the deciding factor in female fitness in North American elk (*Cervus elaphus*; Noyes, Johnson, Dick, & Kie, 2002), as well as several other ungulates (Monteith et al., 2013, 2018). Obviously, gregariousness associated with mating activities may have multiple causations.

## 3 HABITAT, SOCIALITY, DEGREE OF POLYGYNY, AND SEXUAL SIZE DIMORPHISM

**FIGURE 3:**
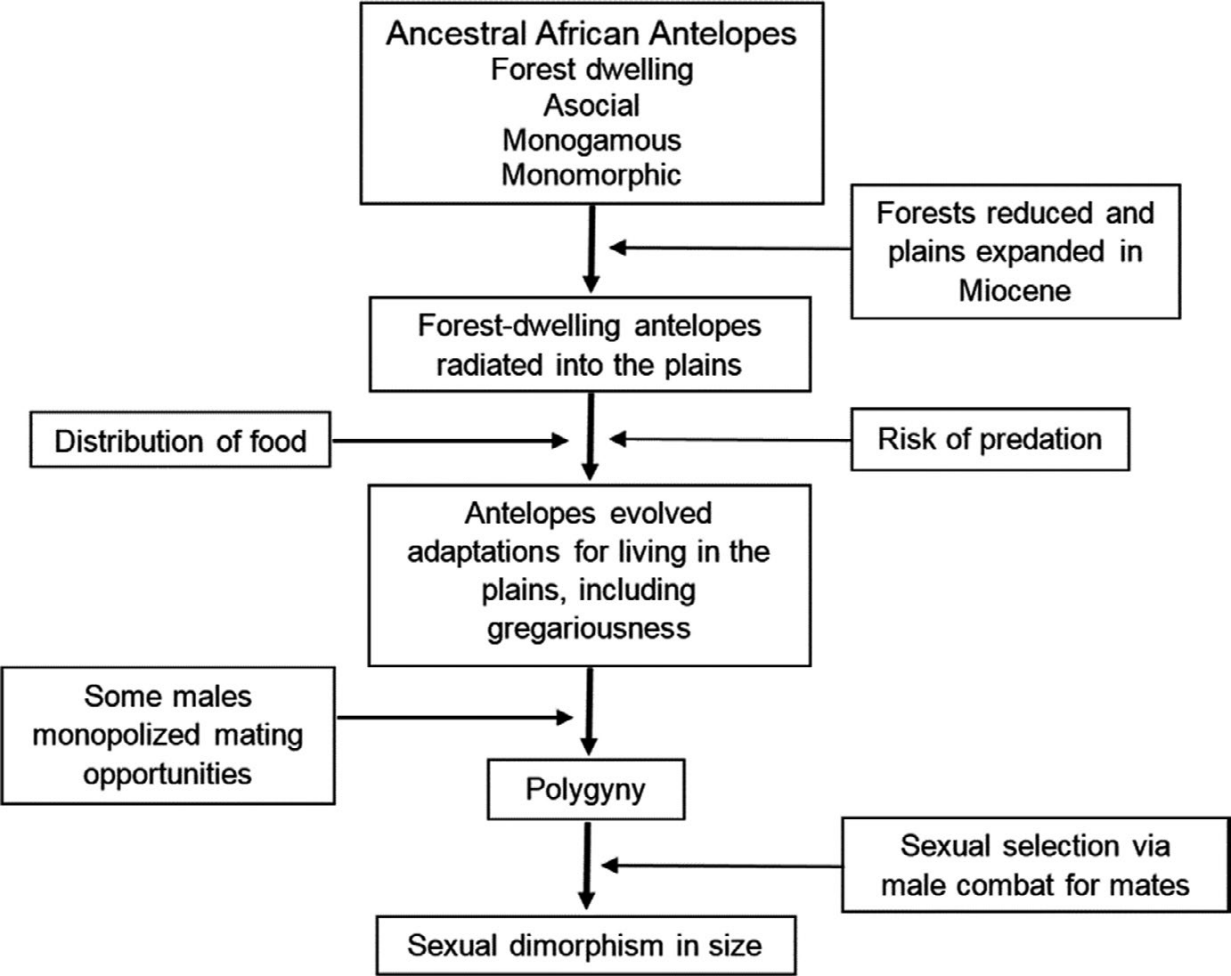
A pattern of evolution for African antelopes (Bovidae) explaining links among habitat, sociality, degree of polygyny, and sexual dimorphism (adapted from Jarman, 1974)

Numerous social, morphological, and environmental factors help shape ungulate mating systems. How are habitat, sociality, degree of polygyny, and sexual dimorphism interrelated, and what role does male–male fighting play in influencing sexual dimorphism in size? Jarman (1974) proposed that ancestors of African antelopes (Bovidae) dwelled in closed habitats (forests) and were unsocial, monogamous, and monomorphic. As grasslands proliferated and forests were reduced during the Miocene, forest‐dwelling antelopes radiated into the plains. Janis (1982) provides paleoecological support for those changes. Plains‐dwelling antelope began acquiring adaptations that allowed them to persist in open habitats, including increased gregariousness. Large social groups provided the opportunity for males to monopolize mating opportunities and favored the evolution of polygyny. The advent of polygynous mating, and accompanying male–male conflicts, likely led to selection for large male body size and, consequently, sexual dimorphism in body size, and the elaboration of horn‐like structures used for varied modes of fighting (Geist, 1966). Indeed, males with the largest horn‐like structures have increased mating success (Kruuk et al., 2002; Markussen et al.., 2019; Vampé et al., 2007). The largest horn‐like structures typically occur in prime‐age individuals (Bowyer, Stewart, et al., 2001; Geist, 1968; Monteith, Schmitz, Jenks, Delger, & Bowyer, 2009). Pérez‐Barbería, Gordon, and Pagel (2002) provide support for the pattern of evolution proposed by Jarman (1974). This general model holds promise for understanding how first habitat, increasing group size, then polygynous mating, and finally sexual size dimorphism sequentially evolved among open‐land ungulates (Figure 3)—factors that may condition the mating system that develops.

Ungulates display huge variation in degree of sexual dimorphism in body size, ranging from monomorphic species to those that are among the most dimorphic of all mammals (Loison, Gaillard, Pelabon, & Yoccoz, 1999; Weckerly, 1998). Weapons and tactics used in male–male combat provide additional insights into degree of sexual dimorphism. Only in those polygynous species in which fighting between males involves wresting or ramming (sensu Geist, 1966; Lundrigan, 1996) would the evolution of sexual dimorphism in size be expected. For instance, horses are monomorphic but are highly polygynous (Berger, 1986; Rubenstein, 1986). Clearly, polygyny is not uniquely linked to sexual size dimorphism in ungulates. Monomorphic collared peccaries (Bissonette, 1982) and vicuña (*Vicugna vicugna*; Cassini, Borgnia, Arzamendia, Benítez, & Vilá, 2009; Franklin, 1983; Koford, 1957) also exhibit polygyny. When agility, speed, and aggressiveness are important (Rughetti & Festa‐Bianchet, 2011), such as in delivering bites, slashing with canines, hooking with horns, or striking with hooves, increased size of a male may not be an advantage in dealing with an opponent. In addition, phylogenetic constraints on the evolution of size dimorphism within particular taxa may exist, or there may be strong concomitant selection for large body size in females (Myers, 1978).

Sexual selection is thought to be the primary cause of sexual dimorphism in mammals (Ralls, 1977), a proposition consistent with hypotheses concerning the role of male–male competition in promoting differences in the body size of sexes among ungulates (Bro‐Jørgensen, 2007). Trivers (1972) proposed, however, that parental investment was the fundamental factor driving sexual selection. The pathway to sexual dimorphism, then, was via the sex making the least parental care to offspring competing most intensively for mates, and therefore experiencing strong sexual selection. Nonetheless, there is a lack of direct paternal care in the monomorphic and monogamous Kirk's dik‐dik (*Madoqua kirki*; Komers, 1996); among ungulates, monogamy is not always linked with paternal investment. For instance, Lukas and Clutton‐Brock (2013) argued convincingly that parental care in mammals was a consequence rather that a cause of monogamy.

**FIGURE 4:**
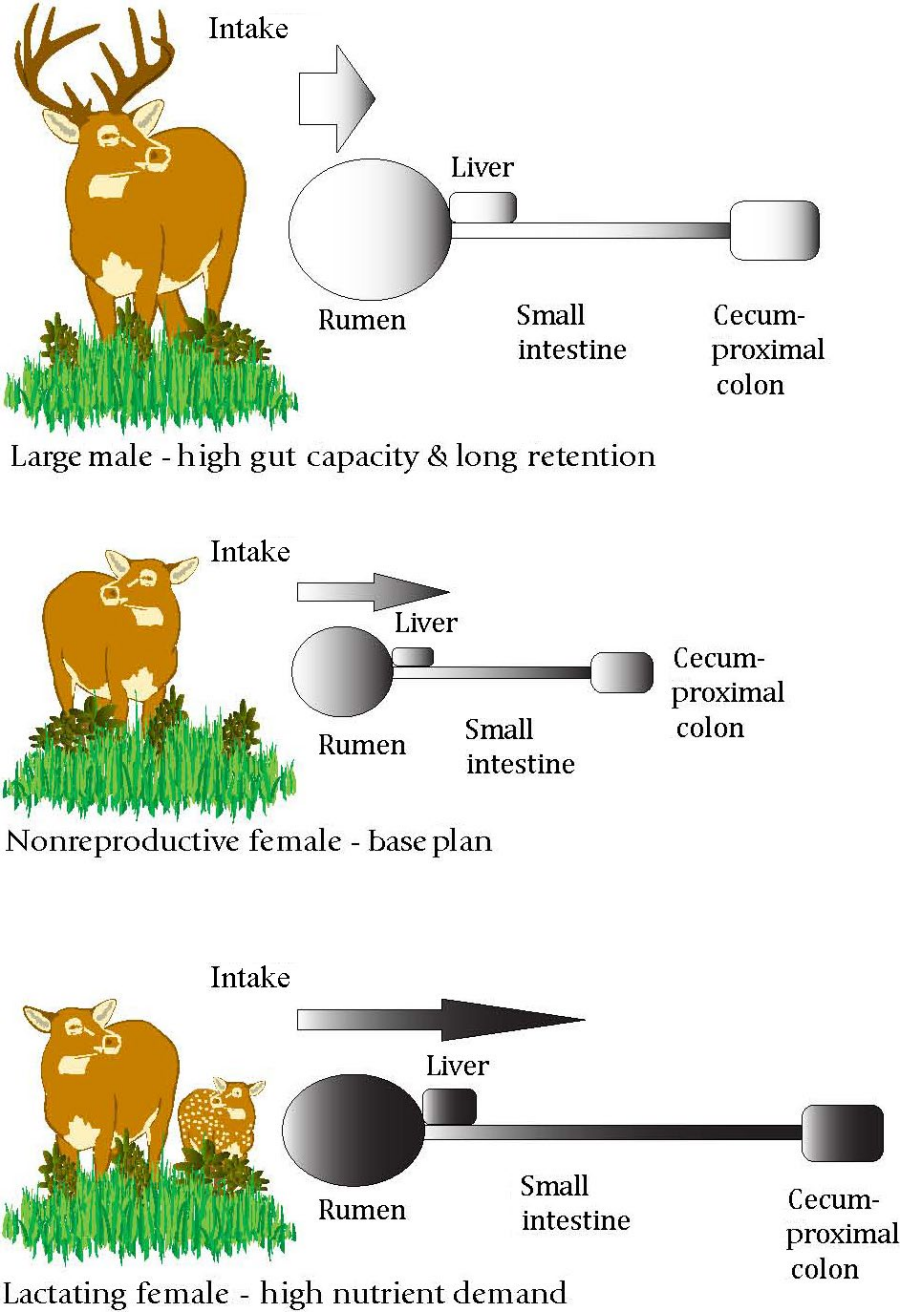
Model of intake and digestive function in nonreproductive females compared with large males and lactating females. Width of arrows reflects amount of food intake, length of arrows indicates rate of digesta passage, and shading indicates density of nutrients in food. Diagrams of digestive tract are stippled to reflect potential changes in fibrosity of food for males and increases in postruminal size and function of lactating females (modified from Barboza & Bowyer, 2000). This figure is modified from the original to include new information (Zimmerman et al., 2006) documenting that the rumen of lactating females is larger and has greater papillae length and width compared with that of nonlactating females (modified from Stewart, Bowyer, & Weisberg, 2011)

Where degree of polygyny is strongly related to the magnitude of sexual size dimorphism in ungulates, instances can occur when a strong feedback mechanism exists that further limits the opportunity for paternal care of young. The sexes of dimorphic ruminants have evolved elaborate differences in their digestive systems to meet essential life‐history requirements—explained by the “gastrocentric hypothesis” (Barboza & Bowyer, 2000). Pregnant females remodel their digestive tracts to accommodate the needs for energy and protein associated with lactation. Maternal females increase the size of the rumen and papillae beyond that of nonreproductive females to enhance digestive capabilities (Zimmerman, Jenks, & Leslie, 2006). The high demand for absorption of nutrients during lactation accelerates production of intestinal and hepatic tissues; those morphological and physiological changes result in a rapid rate of passage for high‐quality forages (Barboza & Bowyer, 2000). Conversely, dimorphic males have an absolutely larger rumen than females, and consume abundant forages that are high in fiber (less digestible); those coarser forages require longer fermentation times in the rumen and consequently have slower rates of passage (Figure 4). Such differences promote spatial separation of males and females by requiring differences in use of foods and sometimes habitats (Barboza & Bowyer, 2000). Predation risk also can promote the degree of sexual segregation, because females and young are more vulnerable to predators than are large males (Bleich, Bowyer, & Wehausen, 1997; Bowyer, 2004; Ciuti, Davini, Luccarini, & Apollonio, 2004). Parturient females may seek refuge in areas minimizing risk of predation but concomitantly sacrificing forage quality (Barten, Bowyer, & Jenkins, 2001; Grignolio, Rossi, Bassano, & Apollonio, 2007). The upshot is that males and females of dimorphic ruminants may be separated spatially for some of the year, especially around the time of parturition, and on occasion in mountain ranges that are >15 km apart (Bleich et al., 1997) or in chiru (*Pantholops hodgsoni*), hundreds of kilometers (Schaller, 1998). The time spent sexually segregated varies among species (Bowyer, 2004) and can be as brief as 2 months in chiru (Schaller, 1998). Under those circumstances, there are few opportunities for males to recognize their offspring and for the development of paternal care. Because of sexual segregation, selection for male–male combat ostensibly is intensified, leading to the evolution of various mating systems.

## 4 MATING SYSTEMS

We offer an overview of ungulate mating systems using specific examples to describe various mating behaviors. We use broad categories to differentiate types of mating systems and subsume many species‐specific subtleties within those categories. We recognize, define, and discuss five broad mating systems in ungulates: pair territories; polygynous resource territories; lek territories; tending bonds; and harems. Other terminology for naming mating systems exists. One was developed for a wide array of organisms (Schuster & Wade, 2003), and another (Clutton‐Brock, 2016) included all species of mammals and was not specific to ungulates (e.g., scramble promiscuity in some rodents). Terms we employ are more specific to ungulates, have been used traditionally, and are widely accepted.

Mating systems may be fluid in some species (Isvaran, 2005); we describe the mating behavior based on the most prevalent system (or multiple systems for some species), and the one most closely related to polygynous mating. For example, in fallow deer (*Dama dama*; Briefer, Farrell, Hayden, & McElligott, 2013), white‐tailed deer (DeYoung, Demarais, Gonzales, Honeycutt, & Gee, 2002), sika (*Cervus Nippon*; Endo & Doi, 2002), and pronghorn (Carling, Wiseman, & Byers, 2003) females occasionally accept copulations from multiple males; those giving birth to multiple offspring may have litters of mixed paternity. This outcome might indicate polyandry or promiscuity, but multiple copulations make up only a small part of mating by females and may relate to fertility insurance (Briefer et al., 2013). Those species generally exhibit primarily polygynous mating, with concomitant intense male–male aggression and sexual dimorphism in size. Moreover, observations of copulations and reproductive success have been established molecularly for polygynous ungulates (Pemberton, Albon, Guinness, Clutton‐Brock, & Dover, 1992). For one or more patterns of mating behavior to be maintained over evolutionary time, those behaviors must be related to reproductive success. In addition, dominant males may selectively mate with “high‐quality” females in American bison (Berger, 1989), white rhinoceros (*Ceratotherium simum*; Rachlow, Berkeley, & Berger, 1998), and mountain goat (*Oreamnos americanus*; Mainguy, Côté, Cardinal, & Houle, 2008), behaviors subsumed within our model of mating systems.

Although we categorize ungulate mating systems with reference to patterns of mating by males, behavior of females also plays an important role in those systems. Female aggression and competition may have important evolutionary consequences (Clutton‐Brock & Huchard, 2013; Fairbanks, 1994; Stockley & Bro‐Jørgensen, 2011). Females may foment fights among males, thereby testing the quality of potential mates. For instance, female pronghorn promote fights among males by leaving a harem and then watching the ensuing combat that this behavior provokes between males; the female then immediately copulates with the winner (Byers, Moodie, & Hall, 1994). Female roe deer make reproductive excursing from male territories during the mating season that may serve as a breeding dispersal tactic (Debeffe et al., 2014). Bowyer et al. (2011) reported that female moose give “protest moans” when being courted by small males, which attracts the attention of the harem master and foments an aggressive encounter. Female moose also may give that vocalization when courted by a large male, which can incite combat between large competitors to gain a mating opportunity, and ostensibly ensure the quality of the successful male (Bowyer et al., 2011). Female topi behave aggressively toward one another for mating opportunities with dominant males on leks (Bro‐Jørgensen, 2002). A broader analysis of the roles of the sexes in mating behavior is likely necessary to more fully understand sexually selected adaptations (Bro‐Jørgensen, 2011a). We believe, however, that those behaviors can be accommodated within the general model that we present, but anticipate that the model could be modified in the future to more fully include the specific roles of female choice in understanding the evolution of mating systems.

Several general patterns in mating behavior of ungulates can be recognized depending upon the environments they inhabit. In mountain‐dwelling bovids, there are mating opportunities for both subadult and adult males. Those polygynous bovids are species with slow body growth; young males are lighter and more agile than older ones. The rugged mountainous environment allows subadults to evade adults and precludes dominants from preventing access of young males to some females. This environment selects strongly for mobility (e.g., for alpine ibex *Capra ibex*; Apollonio, Brivio, Rossi, Bassano, & Grignolio, 2013). Research by Coltman, Festa‐Bianchet, Jorgenson, and Strobeck (2002) on bighorn sheep, and Lovari and Ale (2001) on blue sheep (*Pseudois nayaur*) indicates that subadult males can sire a considerable fraction of newborns, but usually less than older, dominant males. Conversely, in fast‐growing cervids and bovids, which do not inhabit rugged mountainous terrain, mating opportunities often are restricted to adult males that can monopolize females and exclude most young males from access to females (Ciuti & Apollonio, 2016; Pemberton et al., 1992; Wilson et al., 2011). Mating synchrony (Ciuti & Apollonio, 2016), operational sex ratios, and population density (Apollonio, Festa‐Bianchet, & Mari, 1989) also may constrain how many females can be defended effectively.

### 4.1 Territorial systems

Mating systems of ungulates can be categorized broadly into two spatial types: territorial and nonterritorial. Operational definitions of a territory, however, have been elusive (Leuthold, 1977; Maher & Lott, 2000) and not always adequately distinguished from the concept of the home range (Burt, 1943; Leuthold, 1977). Some territories may encompass a small portion of the home range, as reported by Klingel (1974) for large territories of Grevy's zebra (*Equus grevyi*) and African wild ass (*E. africanus*). Conversely, year‐round territories in some small, forest‐dwelling ungulates (Gosling, 1986b; Putman, 1988) may include most of the home range.

What then defines a territory? First, territories used for purposes of mating must be relatively fixed in space and defended against conspecifics. Territory holders are dominant over intruders that attempt to enter their territory, until territory holders become exhausted from rutting activities. Where there are adjacent territories, dominance reversals must occur across territory boundaries, such that each animal is dominant in its own territory, but subordinate in the territory of the adjacent animal (Kitchen, 1974). The criterion of dominance reversals across territorial boundaries eliminates mutually exclusive distributions of animals from our definition of territoriality. Such a distribution might represent discrete home ranges that are not defended, rare or elusive animals where defense and dominance reversals have yet to be observed, or perhaps the development of a mating structure that is intermediate between a nonterritorial and a territorial system. Notably, we do not recognize “moving” territories as a valid concept, because they are not spatially explicit.

#### 4.1.1 Pair territories

Monogamous pair territories tend to occur among small monomorphic species of antelopes that dwell in brush‐dominated or forested areas, including klipspringer (*Oreotragus oreotragus*), dik‐dik, and blue duiker (*Philantomba monticola*; Brotherton & Manser, 1997; Gosling, 1986b); cervids, including muntjacs (*Muntiacus* spp*.*; McCullough, Pei, & Wang, 2000), pudu (*Pudu* spp.; Putman, 1988), and huemul (*Hippocamelus bisulcus*; Povilitis, 1983); and members of Moschidae and Tragulidae, including musk deer (*Moschus moschiferus*; Baskin & Danell, 2003) and probably lesser mouse deer (*Tragulus javanicus*). Males typically defend territories against other males, and females against other females (Putman, 1988), although female dik‐dik (Komers, 1996) and Reeves’ muntjac (*M. reevesi*; McCullough et al., 2000) do not engage in territorial defense. These pair territories are resource‐based and must provide for the needs of the territory holders and their offspring, especially where territories are defended year‐round. Monogamy probably occurs because forage limits the size of groups, but predation risk to females that rely on crypsis to avoid detection also favors small groups (Carranza, 2000). A wide dispersion of females promotes males staying with a single female rather than roaming in search of additional mates (Sandell & Liberg, 1992).

#### 4.1.2 Polygynous resource territories

Male ungulates also defend territories where polygyny is the dominant system. Polygynous resource territories, typically held by a single male, generally encompass important resources such as food, water, or particular types of habitat, and are the most common form of territoriality in polygynous species (Estes, 1974; Gosling, 1986b). Nonetheless, resources available to the territory holder and any females on his territory have been measured infrequently (Balmford, Albon, & Blakeman, 1992; Kitchen, 1974; Rubenstein, 1986). Some species, such as waterbuck (*Kobus ellipsiprymnus*; Spinage, 1982) and white rhinoceros (Owen‐Smith, 1971), hold territories year‐round, whereas others, including pronghorn, defend territories for several months prior to and during the mating season (Kitchen, 1974). Male blue wildebeest (*Connochaetes taurinus*) attempt to defend territories during migration, which only can be held temporarily (Gosling, 1986b). Additional males helping with territory defense has been reported for waterbuck (Wirtz, 1981).

Females ostensibly are drawn to resource territories because of the resources contained therein. In polygynous species, however, male–male competition may limit or mask female choice (Bowyer et al., 2011; Clutton‐Brock & McAuliffe, 2009). Males may attempt to keep females on their territories (and away from those of their adversaries), by aggressive behavior and herding. Females may move among territories to acquire better resources, and males typically have a limited influence on such female movements (Gosling, 1986b; Owen‐Smith, 1971). As Estes (1974) noted, it is inappropriate to use the term “harem” to describe this herding behavior. Harem mating is a nonterritorial system that we discuss later.

Gosling (1986b) postulated four conditions that might lead to the development of a polygynous resource territory: (a) high‐quality and clumped forages, or a heterogeneous supply of foods that would be available for more than one season; (b) a high degree of mating synchrony, with females in estrus for a relatively short part of the year; (c) male familiarity with a small area where an advantageous knowledge of predation risk improves survival; and (d) the cost of resource defense is less than that of numerous aggressive interactions with other males over mating opportunities. These conditions are not mutually exclusive, and more than one may operate simultaneously. Few tests of these conditions have been made for polygynous ungulates.

#### 4.1.3 Lek territories

A lek is an “aggregated male display that females attend primarily for the purpose of fertilization” (Höglund & Alatalo, 1995). Among mammals, lekking has been described in 15 species, nine of which are ungulates, including bovids and cervids (Höglund & Alatalo, 1995; Isvaran, 2005). Explanations for leks, however, are more complex than for other mating systems. Females usually visit the lek and leave it soon after mating, whereas males stay and continue courtship toward other females (Apollonio et al., 2014). The most dominant male usually, but not always, occupies the central territory on the lek (Bro‐Jørgensen, 2011b; Isvaran & Jhala, 2000).

Leks in ungulates can be distinguished from other mating systems according to a number of criteria adapted for ungulates (Bradbury, 1981; Höglund & Alatalo, 1995). The main feature of a lek is that it does not contain substantial resources required by females, except the males themselves, and this pattern clearly distinguishes lek territories from clustered, polygynous resource‐based territories. Ungulate leks usually occur at traditional sites (Apollonio et al., 2014) and are characterized by skewed mating success among males (Apollonio, Festa‐Bianchet, Mari, Mattioli, & Sarno, 1992; Apollonio, Festa‐Bianchet, Mari, & Riva, 1990; Balmford et al., 1992; Ciuti, Cena, Bongi, & Apollonio, 2011). Males often hold permanent territories, and the same male can be located on the same lek territory for days, and even for several consecutive mating seasons (Ciuti et al., 2011).

A number of models have been proposed to explain the evolution of leks in ungulates (Apollonio et al., 2014). The female harassment model (a.k.a., black hole model; Clutton‐Brock, Price, & Maccoll, 1992; Stillman, Deutsch, Clutton‐Brock, & Sutherland, 1996) predicts that the sexual harassment by subadult males leads females to find refuge within a territory held by an adult male, and adult males increase their chance to retain harassed females if they cluster in a lek.

The hotshot model (Beehler & Foster, 1988) predicts that females prefer to mate with an attractive male (hotshot), and less‐attractive males try to parasitize the attractiveness of the hotshot, thereby leading males to cluster in a lek. According to the female preference model (Bradbury, 1981), a.k.a., female bias hypothesis (Isvaran & Ponkshe, 2013), leks should form because females prefer a large clump of males, leading to increased opportunities for mate choice and greater probability of finding a high‐quality mate.

In contrast, the hot spot model (Bradbury & Gibson, 1983; Bradbury, Gibson, & Tsai, 1986) predicts that males should cluster in sites of high female traffic (hotspots), because of increased encounter rates with females. Finally, the predator‐avoidance model (Wiley, 1973) predicts that leks would be favored by reduced risk of predation, because of a dilution effect (Hamilton, 1971), and that females should group in those areas where predation risk is lower. For instance, leks in topi (*Damaliscus lunatus*) and kob (*Kobus kob leucotis* and *K. k. thomasi*) were located where the grass on the savanna was shorter and enhanced visibility reduced risk of predation (Gosling & Petrie, 1990), or perhaps allowed females a greater opportunity to exercise mate choice. According to Höglund and Alatalo (1995) and Apollonio et al. (2014), multiple explanations of lek formation reasonably coexist, and a single factor is unlikely to explain lek evolution in species with contrasting life histories or those living in different ecological contexts.

**FIGURE 5:**
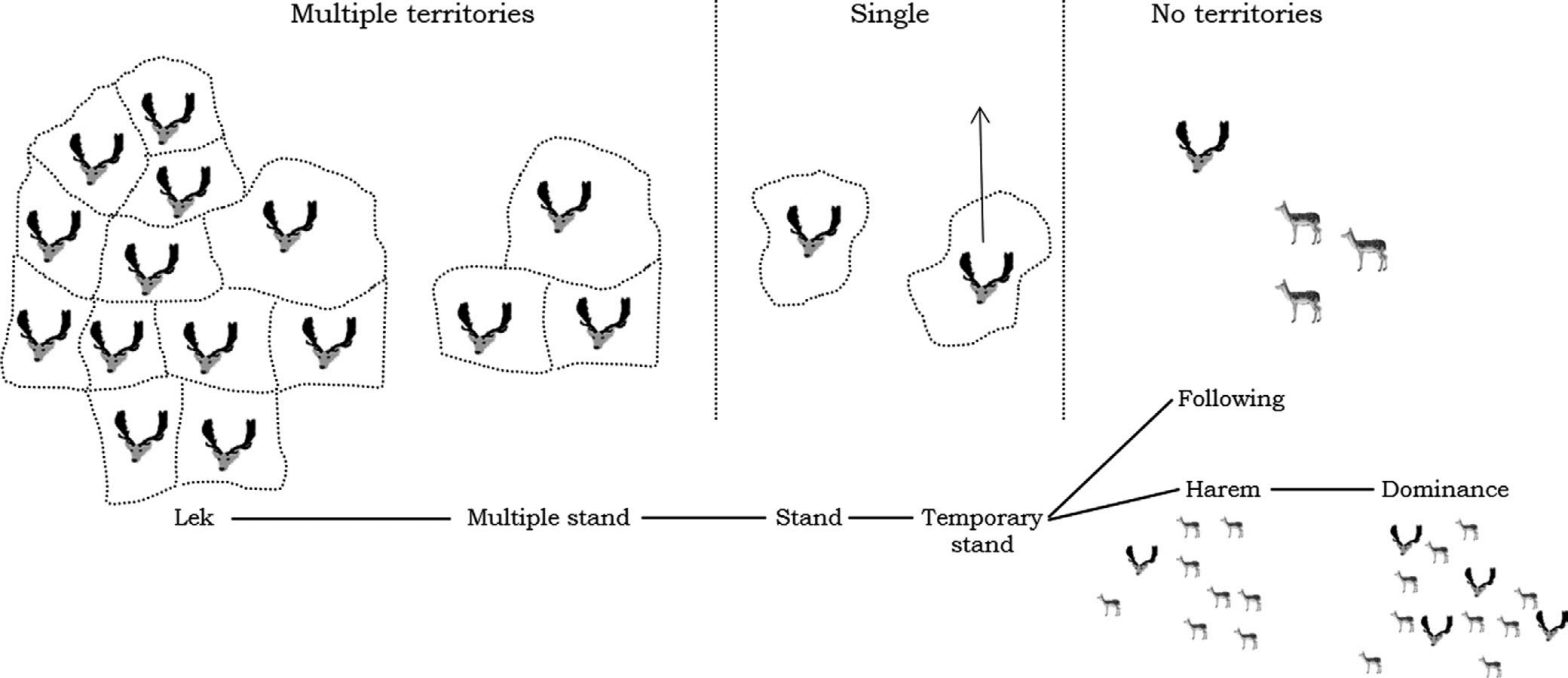
Schematic view of the range of observed variation of fallow deer mating systems (modified from Langbein & Thirgood, 1989). A multiple stand can be distinguished from a lek mainly by the low number of territories (<5)

Many species that mate on leks also display the largest variation in their mating system documented for ungulates, of which fallow deer is the most compelling example (Figure 5; Ciuti et al., 2011; Isvaran, 2005; Langbein & Thirgood, 1989). As suggested by Langbein and Thirgood (1989), the main ecological factors thought to influence occurrence of lekking in ungulates are habitat structure, demographic factors (population density and sex ratio), and behavior of females (home range and grouping patterns).

Resources and habitat structure are thought to influence lekking by affecting female density and distribution (Deutsch, 1994; Gosling, 1986a; Nefdt & Thirgood, 1997). Many studies reported a positive relationship between the occurrence of lekking and high population densities, mainly because males can attract enough females to counterbalance the large costs of defending a lek territory (Balmford, Bartos, et al., 1993; Clutton‐Brock, Green, Hiraiwa‐Hasegawa, & Albon, 1988). Others (Apollonio et al., 1989; Isvaran, 2005), however, noted that local patterns in female distribution (e.g., group size and sex ratio) can be more important than overall population density. For example, one population of lekking fallow deer had moderate population density, but locally high concentrations of females, because of a heterogeneous environment (Apollonio, 1989). Lekking also occurs in species in which females move unpredictably in large groups and have large home ranges (Balmford, Deutsch, Nefdt, & Clutton‐Brock, 1993; Bradbury et al., 1986; Clutton‐Brock, Deutsch, & Nefdt, 1993). If resource or female defensibility is not economical for males because females occur in large groups, at high local densities, or range within wide areas, then clustering of males into a lek is strongly favored (Clutton‐Brock et al., 1993; Gosling, 1991).

### 4.2 Nonterritorial systems

#### 4.2.1 Tending bonds

A tending bond usually is characterized by a dominant male that courts and defends one estrous female at a time, although other strategies exist in this mating system (Hogg & Forbes, 1997; Pelletier, Hogg, & Festa‐Bianchet, 2006). The tending dominant male is usually the largest, mature individual with well‐developed horn‐like structures (Coltman et al., 2002; Hogg & Forbes, 1997; Newbolt et al., 2017). A tending male creates mating opportunities by consorting with a single, estrous female, and preventing other subordinate males from mating with that female by using behavioral gestures and threats, body shielding, and physical attacks—such as pushing, kicking, chasing, and butting the subordinate with horn‐like structures (Hogg, 1984). The tending male continues this aggressive behavior until subordinate males move from the vicinity of the female (Geist, 1971). The dominant, tending male does not restrict the movement of the female and usually copulates with her after some mildly evasive behavior by the female (Hogg & Forbes, 1997). Courtship chases may ensue with subordinate males trailing behind the estrous female and dominant male (Hirth, 1977). Tending is the primary mating system for mountain ungulates, such as mountain goats (Festa‐Bianchet & Côté, 2008) and polygynous ungulates inhabiting densely forested or wooded areas, such a mule deer (*Odocoilius hemionus*), white‐tailed deer (Airst & Lingle, 2019; Hirth, 1977; Kucera, 1976), many subspecies of moose (Altmann, 1959), and Bhutan takin (*Budorcas taxicolor whitei*; J. Berger, personal communication). Tending bonds also occur in some ungulates that form enormous groups such as caribou (*Rangifer tarandus*; Bergerud, 1974; Lent, 1965) and bison (Berger & Cunningham, 1994; Bowyer et al., 2007; Lott, 1974), where groups ostensibly are too large to allow herding of females and successful harem mating (discussed later).

Other strategies in a tending‐bond system are blocking and coursing (sometimes termed roving). These strategies are considered opportunistic, because subordinate males take advantage of mating opportunities, gaining temporary access to copulate with females (Geist, 1971; Hogg, 1984; Pelletier et al., 2006). Blocking involves subordinate males that encourage female movements away from the tending area or that prevent females from traveling in the direction of the tending area until after the females become receptive (Coltman et al., 2002; Hogg, 1984). In this strategy, attempts by the female to escape are blocked by the subordinate male positioning his body, as well as the male threatening and attempting to attack the female (Hogg, 1984; Hogg & Forbes, 1997). Coursing males, often of lower dominance rank, provoke aggressive interactions between a tending male and a female to gain temporary access to the female (Hogg & Forbes, 1997). This tactic involves the lower‐ranking male approaching the tending male and female, and then challenging or attempting to bypass the tending male to copulate with the usually unreceptive female before the tending male can recover (Coltman et al., 2002; Hogg, 1984). Weather conditions also may affect mating strategies by limiting opportunities for coursing males under conditions of deep snow (Apollonio et al., 2013).

The mating strategy employed by a male in a tending‐bond system depends on the social rank of that male. Social rank in male Rocky Mountain bighorn sheep (*O. c. canadensis*) is determined by age, horn size, body mass, and testosterone levels (Hass & Jenni, 1991). Those rankings are linear from dominate to subordinate; therefore, subordinate males often use less conventional tactics to mate with females (Pelletier & Festa‐Bianchet, 2006). In the tending‐bond system of bighorn sheep, one mature large‐horned male that tended females sired 36% of the young in one mating season (Coltman et al., 2002). Although tending males gained higher mating success than coursing males, about 44%–50% of the offspring were sired by males using blocking or coursing tactics (Coltman et al., 2002; Hogg, 1984; Hogg & Forbes, 1997). Additionally, other mating tactics by dominant individuals to enhance their success include “retaliatory copulations” and subsequent sperm competition. After females have copulated with subordinate coursing males, dominants immediately copulate with those females to enhance their reproductive success and thwart that of subordinates (Hogg, 1988).

#### 4.2.2 Harems

Mating in harems is fundamentally different from other nonterritorial systems. In harem mating, usually a single dominant male, termed the “harem master,” attempts to herd and defend a group of females, and mate with them as they come into estrus (Bowyer & Kitchen, 1987; Clutton‐Brock, Guinness, & Albon, 1982; McCullough, 1969). Dominant harem masters take advantage of existing groups of females and tend to move with them while attempting to keep them bunched by herding to prevent females from leaving the harem. Subordinate, “bachelor” males often occur on the periphery of harems and attempt to “sneak” copulations; fights over possession of the harem between bachelor males and the dominant larger harem master are rare (Clutton‐Brock et al., 1982; McCullough, 1969). Not all mating, however, is by dominant males; Bowling and Touchberry (1990) reported that nearly one‐third of young were not sired by harem masters in wild horses. Serious fights over possession of the harem usually occur among opponents of near equal size and dominance rank, especially as harem masters become exhausted from strenuous rutting activities (Bowyer, 1981; Clutton‐Brock et al., 1982; McCullough, 1969). In North American elk, the introduction of domestic cattle (*Bos taurus*) ostensibly disrupted harems and allowed small males to obtain some copulations (Kie et al., 2013).

Harem mating has been described in muskoxen (Gray, 1987; Gunn, 1992; Ihl & Bowyer, 2011), North American elk and red deer (Bowyer & Kitchen, 1987; Clutton‐Brock et al., 1982; McCullough, 1969), fallow deer (Langbein & Thirgood, 1989), sika (Endo, 2009), wild horses (Berger, 1986; Feist & McCullough, 1976), Alaskan moose (*A. a. gigas*; Bowyer et al., 2011), and several species of zebra (Boyd, Scorolli, Nowzari, & Bouskila, 2016; Klingel, 1978). Harem mating for species inhabiting steep and rugged terrain is uncommon, likely because effective herding of females by dominant males would be challenging. Likewise, dense vegetation with associated small groups of females, or too few estrus females, may make the energetics of harem mating unprofitable for large males (Bowyer et al., 2011).

As with other mating systems (Caro & Bateson, 1986; Gross, 1996), alternate mating strategies may occur in harem systems. Indeed, in some bands of wild horses, multimale alliances exist, in which a dominant stallion is assisted by up to five subordinates in harem defense (Feh, 1999; Linklater & Cameron, 2000; Stevens, 1990). Subordinate males are more likely to engage in harem defense against intruders, while the dominant stallion herds females away from those interlopers; reproductive opportunities for subordinates are meager at best, and most alliances are short‐lived (Berger, 1986). Factors promoting multiple‐male alliances in wild horses have been hotly debated (Linklater, Cameron, Stafford, & Minot, 2013); cooperation, reciprocal altruism, and mutualism are no longer considered valid hypotheses. The “limited control” hypothesis (e.g., mate parasitism) has more support than other hypotheses, but does not offer a complete explanation for this phenomenon (Linklater et al., 2013).

## 5 INTERSPECIFIC DIFFERENCES IN MATING SYSTEMS—A CONCEPTUAL MODEL

We integrate tactical and strategic modeling approaches to predict the evolution of ungulate mating systems, which sacrifice some precision to gain a broad grasp of general principles (May, 2001). We include relevant specifics, but keep our model of mating systems simple so it can be interpreted readily (sensu Kokko, 2007). Our intent is to identify important determinants of mating systems, but keep a description of those factors general. We do not quantify parameters within our model, but set forth conditions that can be enumerated, tested, and refined by further research. Indeed, our purpose is to provide a model that includes components that guide future tests of hypotheses concerning ungulate mating systems.

**FIGURE 6:**
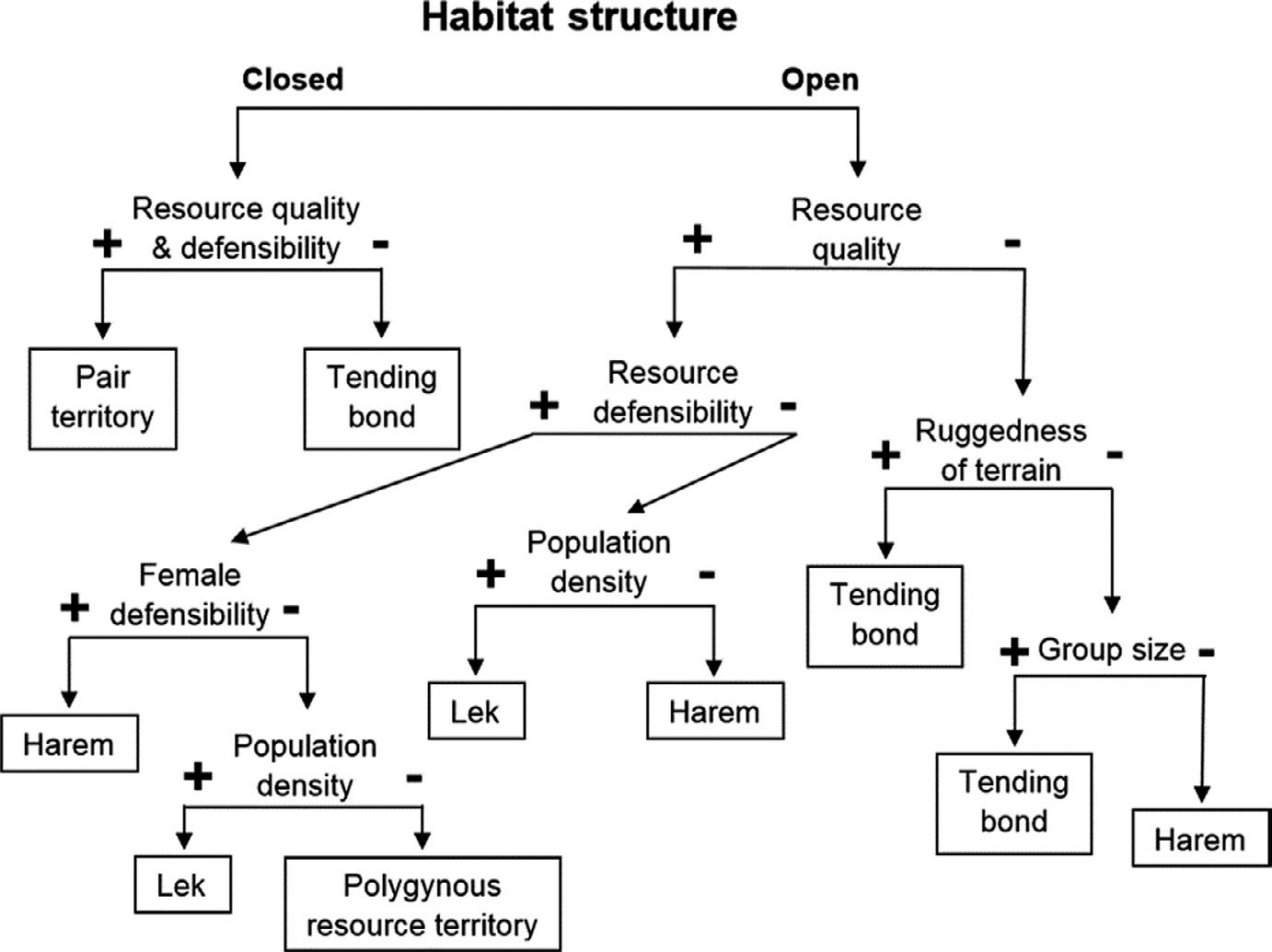
A conceptual model predicting major categories of ungulate mating systems based on environmental and social factors. Examples of specific ungulates are provided in the text. Positive signs (+) indicate greater resource quality, greater ability of males to defend resources, increased ruggedness of terrain, greater ability of a male to defend a female from competitors, and higher density of animals and larger group size. Negative signs (−) indicate the converse of those measures

Considerable variation exists among social systems of ungulates. Those systems are directly and indirectly influenced by aspects of the environment inhabited by the various species, as well as by the nature of the animals themselves. Our broad conceptual model (Figure 6) starts with *Habitat Structure* (including distribution of forages), as modified by risk of predation (open or closed structure) as the initial vairable; as noted previously those are principal determinants of gregariousness in ungulates (Figure 3), and is an appropriate beginning for categorizing mating systems. Although our model is constructed as a series of dichotomies, we recognize that, in reality, a continuum exists for most model components, a point that is particularly germane for habitat structure. We envision closed habitats as dense and expansive forests. Some ungulates may use patches of habitat within forests for mating, but these open areas foster mating systems more typical of ungulates occupying open habitats. For instance, Roosevelt elk (*C. e. roosevelti*) can occur in extensive stands of old‐growth redwood forest (*Sequoia sempervirens*), but gather in forest opening for mating and exhibit a harem system typical of other open‐land species (Weckerly, 2017). Extensive meadows or savanna are examples of open habitat, but can include smaller patches of forests or woodlands. Thus, closed habitat occurs where the forest extends well beyond the average home‐range size of animals. In open habitats, any forest patches would be smaller than the average size of home ranges.

Moreover, Estes (1974) recognized a suite of life‐history traits that were associated with living in open or closed habitats for African antelopes, including diets, responses to danger, body size, type of horns, and degree of sexual dimorphism. Hirth (1977), for example, documented that groups of white‐tailed deer inhabiting dense woodlands in Michigan, USA, were much smaller than those from more open areas in Texas. Likewise, Bowyer, McCullough, et al. (2001) noted that groups of mule deer were smaller in habitats with greater concealment cover, and group size increased as deer moved away from the edge of wooded areas into open meadows. Thus, animals inhabiting closed habitats would be expected to occur in small social groups.

For forest and woodland‐dwelling species, the next delimiting factor of mating systems is *Resource Quality*, which is a comparative term that may be defined with standard measures for forage analyses. *Defensibility *is the ability of an individual to defend clumped resources from competitors. Where resources are of sufficiently high quality (+) and distributed in a manner that would allow their defense (+), the model predicts the development of a *Pair Territory*. Examples would include most small, monomorphic bovids and cervids inhabiting forested ecosystems (Estes, 1974; Putman, 1988). Where resources are of lower quality (−) and not distributed in a defensible manner (−), the model predicts a *Tending Bond* mating system. White‐tailed deer inhabiting dense boreal forests or closed tropical woodlands provide good examples of this outcome (Hirth, 1977). We do not attempt to assess or quantify resource quality and defensibility—doing so across the numerous species of ungulates would be problematic. Our purpose is to propose hypotheses regarding selected environmental variables and their role in the elaboration of ungulate mating systems that can be tested in the future.

Where ungulates occur in more open habitats, and accordingly form larger groups, resource quality again comes into play (Figure 6). If *Resource Quality* is high (+), but *Resource Defensibility* is low (−), then population density becomes a determining factor. Where *Population Density* is comparatively high (+), the prediction is for a *Lek*‐mating system. Fallow deer fit this model nicely (Apollonio et al., 1989; Ciuti et al., 2011; Langbein & Thirgood, 1989). Where population density is lower (−), the model predicts *Harem* mating, such as in North American elk (McCullough, 1969) and red deer (Clutton‐Brock et al., 1982). We offer relative comparisons of population density for simplicity and clarity; more specifics are available in the literature citations.

In open habitats with high *Resource Quality* (+) and *Resource Defensibility* (+), the defensibility of females becomes important (Figure 6). Where *Female Defensibility* (ability of a male to defend a female from potential suitors) is high (+), the prediction is for *Harem* mating; the muskox is a good example (Gunn, 1992). If *Female Defensibility* is low (−), however, population density again plays a role in determining the mating system. Where *Population Density* is low (−), a *Polygynous Resource Territory* is predicted (males can defend territories but have more difficulty in defending the females that pass through them). Examples include white rhinoceros (Owen‐Smith, 1971), pronghorn (Kitchen, 1974), wild horses (*Equus caballus*; Rubenstein, 1986), and puku (*Kobus vardoni*). Collared peccaries also may fall under this prediction, because there is no need to defend females, which share a common dominance hierarchy and territory with males (Bissonette, 1982). Where *Population Density* is high (+), a *Lek* is predicted, which occurs in several subspecies of kob (Buechner & Roth, 1974; Fryxell, 1987) and topi (Bro‐Jørgensen, 2002).

In open habitats with low (−) *Resource Quality*, relative ruggedness and steepness of terrain (which have not been previously considered in the evolution of mating systems) are major determinants (Figure 5). Where *Ruggedness of Terrain* is extreme (+), a *Tending Bond* is expected; this mating system is exhibited by most mountain ungulates (Geist, 1971). The steep, rugged terrain prevents a harem master from herding females effectively. Finally, if relative *Ruggedness of Terrain* is less severe (−), then the size of social groups comes into play. In extremely *Large Groups* (+), harem masters cannot defend or effectively herd large numbers of females, and the prediction is for a *Tending Bond*. Caribou (Bergerud, 1974; Lent, 1965) and American bison (Berger & Cunningham, 1994; Bowyer et al., 2007; Lott, 1974) are extremely gregarious ungulates that mate using a tending‐bond system. Where ungulates are social, but comparatively *Large Groups* do not occur (−), the prediction is for *Harem* mating. Indeed, wild reindeer, which are the same species as caribou but occur in smaller groups, mate in harems (Body, Weladji, Holand, & Nieminen, 2014; Espmark, 1964). We believe our conceptual model (Figure 6) provides a framework for understanding how environmental and social factors interact to determine major types of mating systems in ungulates, and provides a guide for future testing of hypotheses concerning mating systems, and alternative mating tactics in ungulates. Examining exceptions to the model also offers opportunities to gain a further understanding of ungulate mating systems, including phylogenetic constraints on those systems.

Our model for mating systems (Figure 6) is more predictive for some taxonomic groups or species than for others. Forest‐dwelling suids and tayassuids can form exceptionally large groups in densely vegetated areas (Reyna‐Hurtago, Rojas‐Flores, & Tanner, 2009; Sowls, 1984), which is contrary to our model. The giant forest hog (*Hylochoerus meinertzhagen*) and white‐lipped peccary (*Tayassu pecari*) are extreme examples of this gregariousness (Mekonnen, Bekele, & Balakrishnan, 2018; Reyna‐Hurtago et al., 2009). Some species within those families are not well studied, and further research will be necessary to elucidate causes of this gregariousness, although the distribution of clumped resources might provide a reasonable starting point. Similarly, species within the genus *Kobus* often mate on leks, but the black lechwe (*K. leche smithemni*) occurs at high density, but is not a lek‐mating species (Thirgood et al., 1992). This antelope did have territories that superficially resembled leks, but lacked some of the aggressive behaviors expected with lekking behavior (Thirgood et al., 1992). Perhaps this system was intermediate between a polygynous resource territory and a lek. Clearly, more research is needed on similar high‐density populations with polygynous resource territories.

## 6 INTRASPECIFIC VARIABILITY IN MATING SYSTEMS

Female density, dispersion, and females seeking relieve from harassment by subordinate males are important factors in species exhibiting alternative mating systems (Isvaran, 2005). A transition between harem mating and a polygynous resource territory has been described in red deer (Carranza, Alvarez, & Redondo, 1990; Carranza & Valencia, 1999) and wild horses (Rubenstein, 1986). Byers and Kitchen (1988) also reported a shift away from a territorial system to harem mating in pronghorn, ostensibly the result of a shifting age structure of males. Corlatti, Caroli, Pietrocini, and Lovari (2013) noted that chamois exhibited both territorial and nonterritorial systems.

**FIGURE 7:**
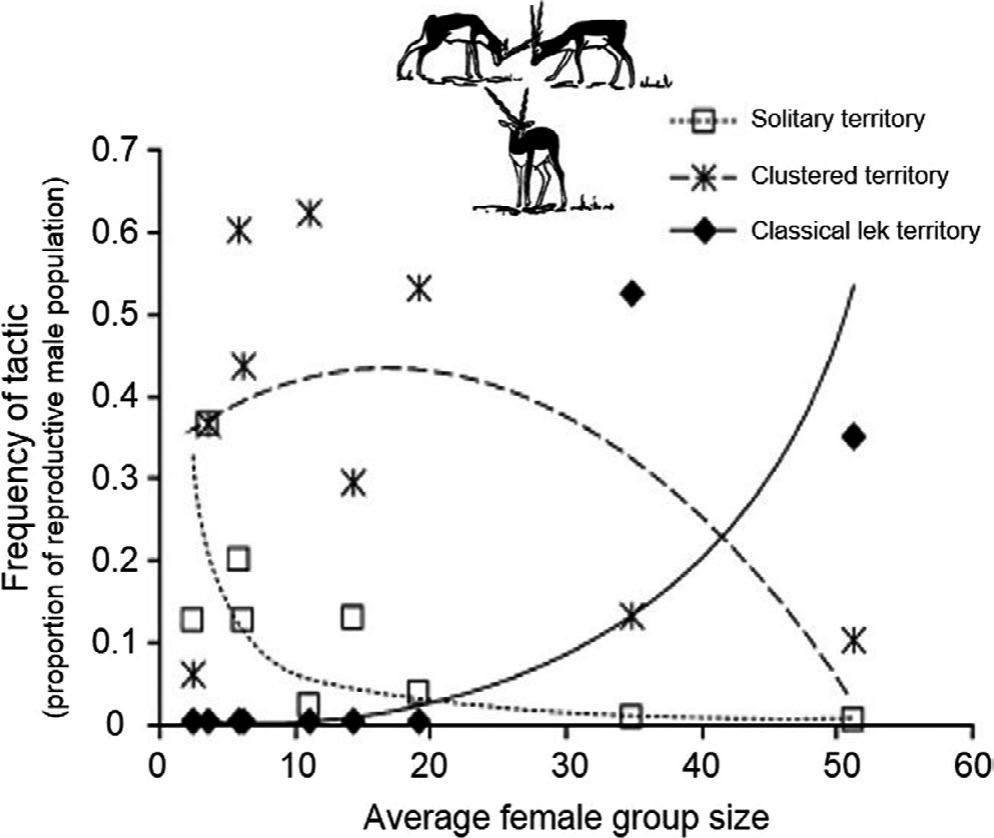
Relative frequencies of male mating systems in relation to local female numbers (female group size) across nine blackbuck populations (from Isvaran, 2005)

Isvaran (2005) described the relative frequency of mating systems in blackbuck across nine populations (Figure 7). The success of males following a particular mating strategy likely changed with the local density of females, with a higher success obtained by older males engaging in high‐risk tactics, and a lower success for younger males following a low‐risk tactic (Isvaran, 2005). Where moose inhabit more open terrain and occur in larger groups (Molvar & Bowyer, 1994), a harem mating system may occur during the first rutting period (Bowyer et al., 2011). Nonetheless, as the mating season progresses into a second rut, when mating groups are smaller because many females conceived during their first estrus, the system changes from a harem to a tending bond (Bowyer et al., 2011). Similarly, male reindeer begin rut by employing harem mating and switch to a tending bond as the mating season wanes (Weladji, Body, Holand, Meng, & Nieminen, 2017). Consequently, changes in mating systems occur within species, but also within the same population for some species, and even during the same mating season, indicating potential effects of both social and environmental conditions on the evolution of mating systems.

## 7 CONCLUSIONS

With orders as diverse as Cetartiodactyla and Perissodactyla, some phylogenetic constraints on ungulate mating systems likely exist. Nonetheless, similar mating systems have evolved across diverse taxa. Pair territories occur in the Cervidae, Bovidae, Moschidae, and probably Tragulidae. Polygynous resource territories are known for the Antilocapridae, Bovidae, Cervidae, Equidae, Hippopotamidae, and Rhinocerotidae. Among ungulates, leks are confined to the Bovidae and Cervidae, whereas nonterritorial systems extend across orders. Tending bonds occur among Bovidae, Cervidae, and probably other forest‐dwelling species that are not territorial. Finally, harems have been described for the Antilocapridae, Bovidae, Cervidae, and Equidae. The similarity of ungulate mating systems across taxonomically diverse families indicates a suite of environmental and social factors likely play a major role in those outcomes.

Ungulates exhibit variation in mating that exists among and within species, which is affected by predation, availability of resources (food and mates), and habitat structure. Our conceptual model provides a basis for synthesizing how environmental and social factors interact to determine the major types of mating systems in ungulates. Our model also provides a useful framework for future tests of the role of both ecological and social conditions in influencing the social systems of ungulates. Our approach is timely and important; mating systems may have demographic consequences for species (McDonald, 2000), with implications for the conservation of these unique mammals (Hogg, 2000).

## CONFLICTS OF INTEREST

The authors declare no competing interests.

## AUTHOR CONTRIBUTION

**Terry Bowyer:** Conceptualization (equal); Writing‐original draft (lead); Writing‐review & editing (equal). **Dale R. McCullough:** Conceptualization (equal); Writing‐original draft (supporting); Writing‐review & editing (equal). **Janet L Rachlow:** Conceptualization (supporting); Validation (equal); Writing‐original draft (equal); Writing‐review & editing (equal). **Simone Ciuti:** Conceptualization (equal); Validation (equal); Writing‐original draft (equal); Writing‐review & editing (equal). **Jericho C. Whiting:** Conceptualization (equal); Validation (supporting); Writing‐original draft (equal); Writing‐review & editing (equal). 

## Data Availability

No data set was used in this manuscript.
